# Snare-assisted submucosal tunneling for resection of esophageal schwannomas: Case Report

**DOI:** 10.3389/fmed.2025.1580999

**Published:** 2025-04-09

**Authors:** Ximei Cao, Jiangtao Li

**Affiliations:** ^1^Department of Gastroenterology, Jiujiang City Key Laboratory of Cell Therapy, Jiujiang No.1 People's Hospital, Jiujiang, China; ^2^Department of Gastroenterology, Jiangxi Provincial Key Laboratory of Digestive Diseases, Jiangxi Clinical Research Center for Gastroenterology, Digestive Disease Hospital, The First Affiliated Hospital, Jiangxi Medical College, Nanchang University, Nanchang, China

**Keywords:** esophageal schwannoma, snare-assisted traction, submucosal tunneling endoscopic resection (STER), endoscopic resection, case report

## Abstract

Esophageal schwannoma is a rare type of esophageal tumor that presents significant challenges in resection, particularly when large and irregularly shaped. This case report highlights the successful removal of a large esophageal schwannoma using a novel technique that combines submucosal tunneling endoscopic resection (STER) with modified snare-assisted external traction. A 68-year-old male with dysphagia was diagnosed with a 35 mm × 20 mm × 15 mm esophageal schwannoma. Due to the tumor’s size and consistency, traditional resection methods proved difficult. The snare-assisted traction technique, applied within the submucosal tunnel, provided optimal tension and improved visualization, enabling en bloc resection. The patient recovered well postoperatively, and follow-up endoscopy confirmed complete healing. This novel traction technique is safe and effective for the resection of large esophageal submucosal tumors (SMTs), offering enhanced procedural efficiency and better outcomes in complex cases. However, the clinical utility of this technique requires confirmation through prospective studies with adequate sample sizes and long-term outcome assessments.

## Introduction

Schwannoma is a peripheral nervous system tumor originating from Schwann cells, typically referring to a benign, slow-growing neoplasm (1). While this type of tumor may develop in any anatomical site, its occurrence in the gastrointestinal tract is uncommon, with esophageal involvement being particularly rare (2–5). Esophageal schwannoma is a sporadic and rare esophageal submucosal tumor (SMT), accounting for less than 1% of all esophageal tumors (6, 7). They are usually located in the upper and middle esophagus and predominantly affect middle-aged women, more commonly found in Asian populations (5, 8). Due to the lack of characteristic imaging features in esophageal schwannomas, preoperative diagnosis is considered challenging, and confirmation is typically made after resection. While most cases are benign, malignant potential exists (9, 10). If detected early and treated with appropriate surgical resection, patients generally achieve a favorable survival prognosis.

Early-stage cases are typically asymptomatic. However, when symptoms such as dysphagia appear, the tumors are usually diagnosed at a larger size, complicating treatment. The standard treatment for esophageal schwannomas is surgical resection, traditionally performed via open surgery or, more recently, using endoscopic techniques (11–13). However, large or irregularly shaped tumors often pose significant challenges to conventional endoscopic approaches, such as endoscopic submucosal dissection (ESD) or STER. For tumors smaller than 3 cm, endoscopic resection is generally preferred, whereas those larger than 3 cm are mostly treated with open surgery (5, 11, 14). If endoscopic resection (e.g., ESD or STER) is attempted for larger tumors, the procedure tends to be time-consuming and often requires full-thickness resection (12, 15). Traditional traction techniques (e.g., using clips and dental floss), commonly reported in gastrointestinal ESD, are typically employed to retract the edges of mucosal lesions (16–18). However, the resection of submucosal tumors within the esophageal tunnel requires preservation of mucosal integrity, rendering conventional traction techniques unsuitable for STER procedures. To address this limitation, we developed a modified snare-assisted traction technique combined with esophageal STER, which, to our knowledge, has not been previously reported. Here, we describe the successful resection of a large esophageal schwannoma using this innovative snare-assisted traction technique during STER.

## Case description

**Figure 1:**
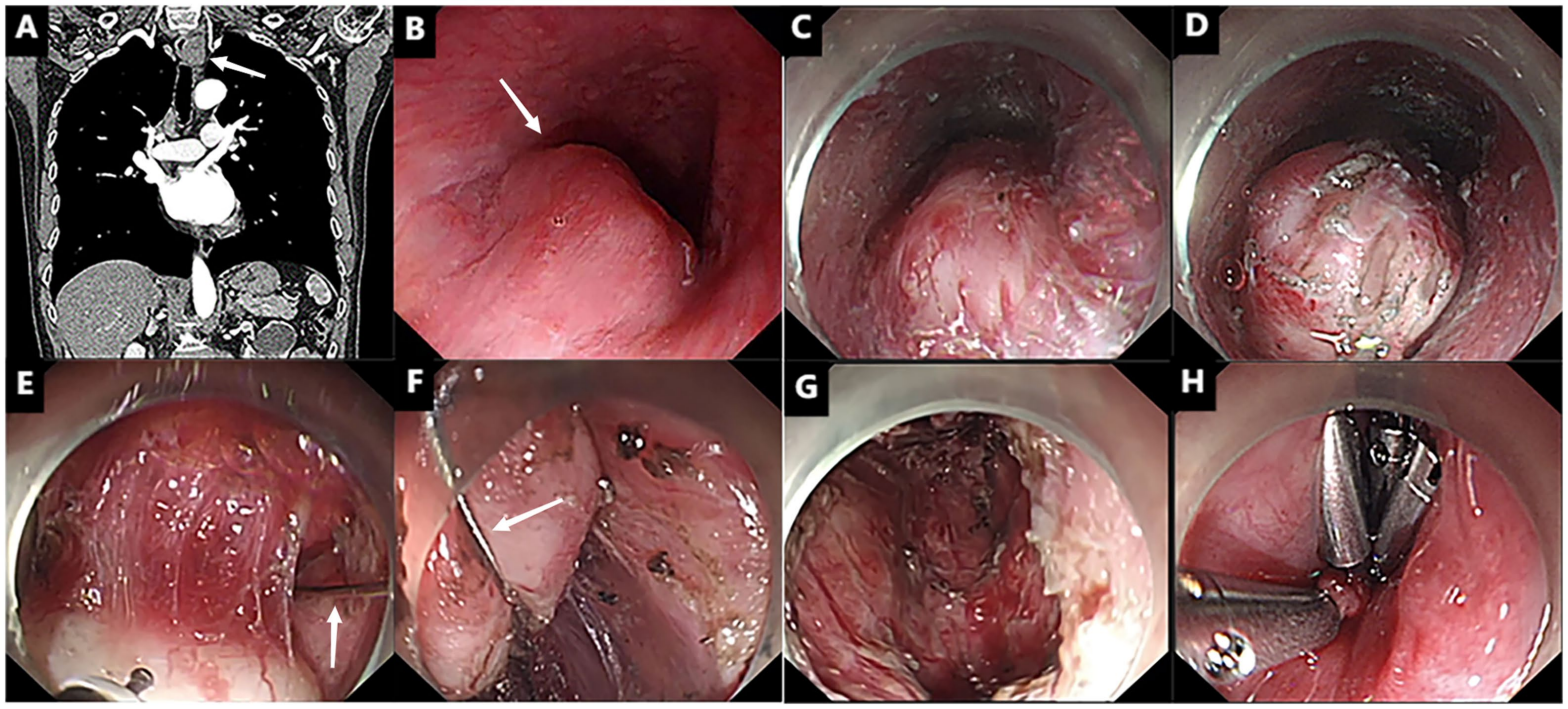
Snare-assisted external traction for STER. **(A,B)** A mass is observed in the upper esophagus (as indicated by the white arrows). **(C,D)** Following the creation of a submucosal tunnel, a large white tumor was revealed (as indicated by the white arrows). **(E,F)** With the assistance of a snare for traction, the tumor was successfully lifted. **(G,H)** The post-endoscopic wound surface was closed using titanium clips.

**Figure 2:**
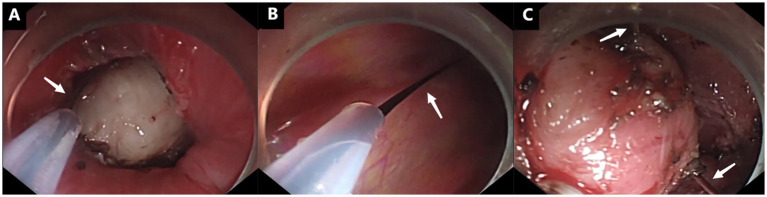
Snare-assisted traction procedure (as indicated by white arrows). **(A)** The snare was looped around the tumor mass within the submucosal tunnel. **(B)** The snare handle was severed, and its outer sheath was removed by the assistant. **(C)** The tumor was lifted by the snare traction device.

In August 2024, a 68-year-old male with a history of progressive dysphagia was admitted to our hospital for evaluation. Computed tomography (CT) revealed a well-defined, mildly enhancing mass located in the upper esophagus (Figure 1A). Endoscopic examination showed a smooth, elevated lesion with poorly defined borders, consistent with a submucosal tumor, approximately 18 cm from the incisors (Figure 1B). However, an endoscopic biopsy could not definitively determine the nature of the tumor. Preoperative endoscopic ultrasound raised the possibility of a leiomyoma. Given the tumor’s size and its firm consistency, traditional endoscopic resection methods, such as ESD, proved challenging. The decision was made to proceed with STER, utilizing the snare-assisted traction technique to facilitate tumor resection. A blended electrosurgical mode (coagulation at 35 W and cutting at 50 W) was employed, with alternating use of a Dual knife and insulated-tip (IT) knife during the procedure. Following creation of the submucosal tunnel, the tumor was adequately exposed (Figures 1C,D). Despite maximal dissection, limited visibility and difficulty accessing the tumor’s deep base posed significant challenges. To address these issues, the snare was introduced into the tunnel through the endoscope, where it first looped around the tumor mass (Figure 2A). Subsequently, the snare handle was severed, and the assistant removed its outer sheath (Figure 2B). Finally, the retained wire was secured with a hemostat to fashion a transoral traction device, providing the necessary tension to lift and pull the tumor (Figure 2C), thereby enhancing tissue manipulation and visualization of the submucosal structures (Figures 1E,F). The procedure mandates strict adherence to safe snare handling principles: First, all traction maneuvers must be conducted under real-time endoscopic visualization. Second, perfect coordination between the operating surgeon and assistant is imperative. Third, tension application should follow a controlled, incremental approach. This triad of precautions effectively reduces the incidence of iatrogenic complications, particularly surrounding tissue damage, mucosal layer violation, and procedure-related perforations (Figures 1G,H).

**Figure 3:**
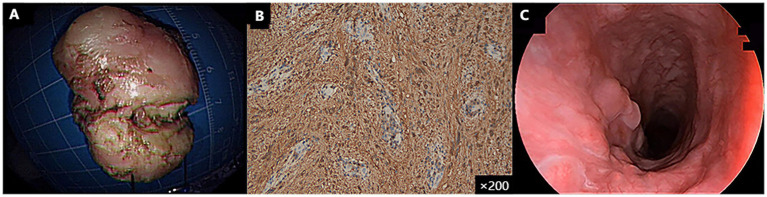
**(A)** An irregularly—shaped solid mass, measuring 35 mm × 20 mm × 15 mm, was resected. **(B)** Based on the positive expression of S100 in the immunohistochemistry examination, it was diagnosed as a rare esophageal schwannoma. **(C)** Three-month follow-up gastroscopy showed good wound healing.

With this traction technique, the tumor was successfully resected en bloc, and the specimen measured 35 mm × 20 mm × 15 mm (Figure 3A). Histopathological analysis confirmed the diagnosis of esophageal schwannoma, with positive S100 immunohistochemical staining (Figure 3B). The total procedure duration was 150 min with an estimated blood loss of 20 mL. The patient tolerated the procedure well and had no immediate postoperative complications. Oral intake was initiated with liquids at 72 h postoperatively, followed by gradual advancement to soft diet. The patient was discharged on postoperative day 5. A follow-up endoscopy **3** months later showed complete healing of the esophageal mucosa with no signs of recurrence (Figure 3C). Further surveillance endoscopies and CT are scheduled at 6 and 12 months postoperatively to evaluate long-term outcomes and recurrence risk.

## Discussion

Schwannomas are rare benign tumors that can occur in the gastrointestinal tract, with the mediastinum being the most common site, while esophageal involvement is relatively infrequent (5). Clinically, it has a high rate of misdiagnosis. It is diagnosed through histology and immunohistochemistry, typically showing spindle cells, S100 positivity, and negative smooth muscle markers (19). Schwannomas generally show poor response to radiotherapy or chemotherapy, and surgical resection is usually the primary treatment (20). When esophageal schwannomas cause symptoms, it typically indicates that the tumor has grown significantly, which increases the difficulty of resection. In the past, esophageal schwannomas were often managed through open surgical approaches, but endoscopic techniques have become increasingly preferred due to their minimally invasive nature. To minimize surgical trauma, we opted for an endoscopic approach.

STER has proven to be an effective alternative for treating esophageal SMTs (21). However, However, technical challenges arise when resecting large (>3 cm) or irregularly shaped lesions. Yuan et al. documented a case involving a 4 cm schwannoma where conventional STER proved inadequate due to tumor bulk, rich vascularity, and limited tunneling space, necessitating conversion to endoscopic excavation with partial full-thickness resection (12). Similarly, Mu et al. reported a 45 × 35 × 31 mm schwannoma resection requiring 5 h of operating time, demonstrating the exponential time increase with larger tumors (15). While snare or dental floss-clip traction techniques have been well-documented in gastric endoscopic mucosal resection (EMR) and ESD (22–24), their application for submucosal tumor dissection within the esophageal tunnel remains technically challenging. To address this limitation, we developed a modified snare system specifically for use in esophageal STER—a technique that, to our knowledge, has not been previously reported.

One of the key challenges in implementing the snare-assisted traction technique is its technical complexity. Due to the narrow space within the esophageal tunnel, the procedure often requires modifications to the snare device, such as cutting off the handle and using the retained wire for external traction. This modification demands a high level of expertise and specialized skills. Inexperienced endoscopists may find it difficult to apply the technique correctly, which could lead to complications such as improper tension or damage to surrounding tissues. Proper training and experience are essential to ensure the safety and efficacy of the procedure, as improper use of the technique could lead to unnecessary complications.

The successful application of this technique expands endoscopic treatment options for large esophageal submucosal tumors. Future studies should explore its utility for other esophageal neoplasms (e.g., leiomyomas or gastrointestinal stromal tumors) and assess feasibility in different anatomical locations (e.g., stomach or colon). Multicenter, large-scale clinical trials will be essential to validate long-term efficacy and safety.

## Data Availability

The original contributions presented in the study are included in the article/supplementary material, further inquiries can be directed to the corresponding author.
